# Leaf silica concentration in Serengeti grasses increases with watering but not clipping: insights from a common garden study and literature review

**DOI:** 10.3389/fpls.2014.00568

**Published:** 2014-10-21

**Authors:** Kathleen M. Quigley, T. M. Anderson

**Affiliations:** Department of Biology, Wake Forest UniversityWinston-Salem, NC, USA

**Keywords:** grass, grazing, silica, defoliation, induced defense, herbivory, phytoliths

## Abstract

Grasses (Poaceae) lack the complex biochemical pathways and structural defenses employed by other plant families; instead they deposit microscopic silica (SiO_2_) granules in their leaf blades (i.e., phytoliths) as a putative defense strategy. Silica accumulation in grasses has generally been considered an inducible defense; other research suggests silica accumulation occurs by passive diffusion and should therefore be closely coupled with whole plant transpiration. We tested the hypothesis that grasses increase leaf silica concentration in response to artificial defoliation in a common garden study in the Serengeti ecosystem of East Africa. Additionally, a watering treatment tested the alternative hypothesis that leaf silica was largely driven by plant water status. Leaf silica content of two dominant C4 Serengeti grass species, *Themeda triandra* and *Digitaria macroblephara*, was quantified after a 10-month clipping × water experiment in which defoliation occurred approximately every 2 months and supplementary water was added every 2 weeks.* Themeda* had greater silica content than *Digitaria*, and *Themeda* also varied in foliar silica content according to collection site. Clipping had no significant effect on leaf silica in either species and watering significantly increased silica content of the dominant tall grass species, *Themeda*, but not the lawn species, *Digitaria*. Our data, and those collected as part of a supplementary literature review, suggest that silicon induction responses are contingent upon a combination of plant identity (i.e., species, genotype, life history limitations) and environmental factors (i.e., precipitation, soil nutrients, grazing intensity). Specifically, we propose that an interaction between plant functional type and water balance plays an especially important role in determining silica uptake and accumulation.

## INTRODUCTION

Plants have two general and non-mutually exclusive adaptive strategies to cope with herbivory: tolerance and resistance (Mauricio et al., 1997). Tolerance implies the capability to survive and regrow following damage by herbivores. In contrast, resistance strategies serve to prevent or reduce damage by herbivores and may entail direct resistance via production of toxic or distasteful secondary metabolites or indirect resistance by avoiding herbivores altogether through reduced apparency (Feeny, 1976). From a resource allocation standpoint, defensive structures (i.e., spines, thorns) and phytochemicals (i.e., tannins) associated with direct herbivore resistance are costly because they require energy and nutrient resources that could otherwise be invested in growth or reproduction. Thus, while some defenses are constitutively expressed, many other plant defenses are induced only after damage is experienced as a way to reduce their costs (Agrawal and Rutter, 1998; Arimura et al., 2000; Massey et al., 2007).

Some plant groups face relatively intense or frequent herbivory and utilize both tolerance and direct resistance strategies. For example, many species of grasses (family *Poaceae*) experience herbivory in the form of defoliation by large-bodied mammalian grazers, resulting in frequent and significant tissue loss (Gibson, 2009). Due to rapid regrowth from a basal intercalary meristem following defoliation, grasses are highly resilient to grazing stress and are typically considered grazing “tolerators” (Stebbins, 1972; McNaughton, 1979; Oyarzabal and Oesterheld, 2009). On the other hand, grasses also utilize both secondary chemicals, e.g., phenolics (Schaller et al., 2012), and structural components, e.g., microscopic deposits of solid silica termed phytoliths (Ma and Yamaji, 2006), to deter herbivores. Evidence suggests that phytoliths have been present in grasses since their early evolution, as long ago as the Late Cretaceous (Prasad et al., 2005; Strömberg, 2011). Accumulation of silica phytoliths has been considered the main defensive strategy of grasses (Coughenour, 1985), as they can amass relatively large amounts of silica and lack chemical defenses as compared to dicotyledonous plants. However, other than grasses, silica accumulation occurs primarily in ancient plant groups such as mosses, ferns, and horsetail (Hodson et al., 2005), and this fact, along with the observation that tooth enamel is considerably harder than phytoliths (Sanson et al., 2007) raises questions about the efficacy of silica as a deterrent of large-bodied mammalian grazers. Among angiosperms, Poales (the group containing grasses) are the principal silica accumulators, with wetland Gramineae accumulating up to 15% dry weight silica (Epstein, 1999). Biogenic silica is assimilated when roots absorb silicic acid (Si(OH)_4_) from soil water, and solid amorphous silica (SiO_2_) bodies precipitate in target cells of the epidermis as transpiration occurs (see Rudall et al., 2014). Phytoliths leave behind a three-dimensional impression of the cells that they inhabit and, because of their diagnostic nature, are often used in paleoecological reconstruction (Piperno and Pearsall, 1998).

Both active and passive mechanisms for silica uptake have been documented within the plant kingdom; active exclusion has also been observed in dicotyledonous angiosperms (Jones and Handreck, 1969). Passive uptake allows grass roots to absorb silicon (Si) in its aqueous form, Si(OH)_4_, from the soil solution and implies limited control over silica accumulation, with stomatal conductance largely determining foliar silica content (Sangster et al., 2001). While early researchers assumed that passive uptake, in which tissue silica concentration increases as a function of transpirational water loss (Sangster and Parry, 1970; Street, 1974), was the predominant mechanism for silica accumulation, several lines of evidence have since suggested that active uptake is also often involved. For example, specialized Si eﬄux transporters have been identified in rice (Ma et al., 2006, 2007), maize, and barley (Mitani et al., 2009) amongst other species, and gene expression of these transporters is positively correlated with Si absorption from the soil solution, implying an energetic cost associated with Si transport in these high-Si accumulators. Grass foliar silica content exhibits high intra- and interspecific variation (Hodson et al., 2005), often in ways that are correlated with disturbance regimes such as grazing and fire frequency (Melzer et al., 2009). These lines of evidence suggest that active Si transport may be an important, and prevalent, mechanism for silica accumulation within Poaceae. An active mechanism could prove especially beneficial if plants are able to respond to herbivory by increasing silica uptake and silica is an effective defense mechanism, as several studies have suggested. For example, high silica content in plant tissues interferes with digestion (Massey and Hartley, 2009), is unpalatable as forage (Gali-Muhtasib et al., 1992; Cotterill et al., 2007) and reduces growth rates of small-bodied mammals (Massey and Hartley, 2006). Consistent with the putative effects of silica on extant herbivores, paleontological research suggests that grazing Hadrosaurid dinosaurs evolved the most complex dentition known to date, at least in part due to a high silica diet (Erickson et al., 2012).

Inducible uptake of silica, via an active metabolic mechanism, may also prove advantageous for coping with several types of abiotic stress. In addition to its effects as a documented anti-herbivore compound, biogenic silica is known to alleviate plant stressors such as heavy metals (Galvez et al., 1987), pathogenic pests (Fauteux et al., 2006; Heine et al., 2007), salinity (Zhu et al., 2004), high temperatures, and drought (Agarie et al., 1998). Thus, it is logical that uptake, if active, may also be inducible under particular environmental conditions. Water stress affects plants ubiquitously, and it appears that silica accumulation alleviates the deleterious effects of drought for grasses. Agricultural studies indicate that soil Si fertilization decreases stomatal conductance, and thus transpirational water loss (Street, 1974; Gao et al., 2006), and enhances the stability of rice cell membranes exposed to drought (Agarie et al., 1998).

The beneficial effects of plant biogenic silica, together with the apparently low cost of assimilating and using silica as a defense have left some to conclude that silica has largely been overlooked as a vital element for stress tolerance (Cooke and Leishman, 2011a). Our study had two goals relating to the ecological significance of plant silica. First, we aimed to quantify the response of leaf silica accumulation to interactions between water availability and defoliation in two African C4 grass species, one (*Themeda triandra*) a caespitose “bunch” grass and the other (*Digitaria macroblephara)* a short “lawn” grass. To achieve this goal we conducted a common garden study in Serengeti National Park, a grazer-dominated ecosystem in East Africa, in which we manipulated defoliation and water-availability over a 10-month growing period and quantified the silica responses of our two focal species. Previous research on similar grasses from the Serengeti demonstrated that grasses are capable of up-regulated silica concentrations in response to herbivory (McNaughton and Tarrants, 1983). Consequently, we hypothesized that defoliation would induce silica uptake in our two study species. In a different study of African savanna grasses, defoliated bunch grasses exhibited greater stomatal conductance and transpiration rates than defoliated lawn grasses (Anderson et al., 2013). Following these observations, we proposed a second prediction that, if silica uptake is directly linked to transpiration rate as previously reported, the bunch grass *T. triandra* would exhibit a greater silica induction following defoliation than would the lawn grass, *D. macroblephara*. Moreover, if silica accumulation is tightly coupled with transpiration rates, we expected to observe increased silica accumulation under conditions of higher soil moisture. Alternatively, if no relationship between soil moisture and silica concentration was observed, this suggests that active, energy-dependent silica uptake mechanisms may predominate in these species. Finally, we explored the possibility that the silica content of grasses was driven, at least in part, by their growing environment (soils, climate, herbivory, etc.). Thus, we also tested whether the site of collection was significantly related to silica variation among grasses in our common garden study.

Our second aim was to understand the results of our study in relation to a literature review of all studies reporting defoliation effects on leaf silica concentration in grasses. Our goal here was to search for a broad ecological consensus of graminoid responses to leaf defoliation and if possible, establish generalities about grass–grazer interactions and the induction of silica plant defense.

## MATERIALS AND METHODS

### COMMON GARDEN STUDY

#### Study system

**FIGURE 1:**
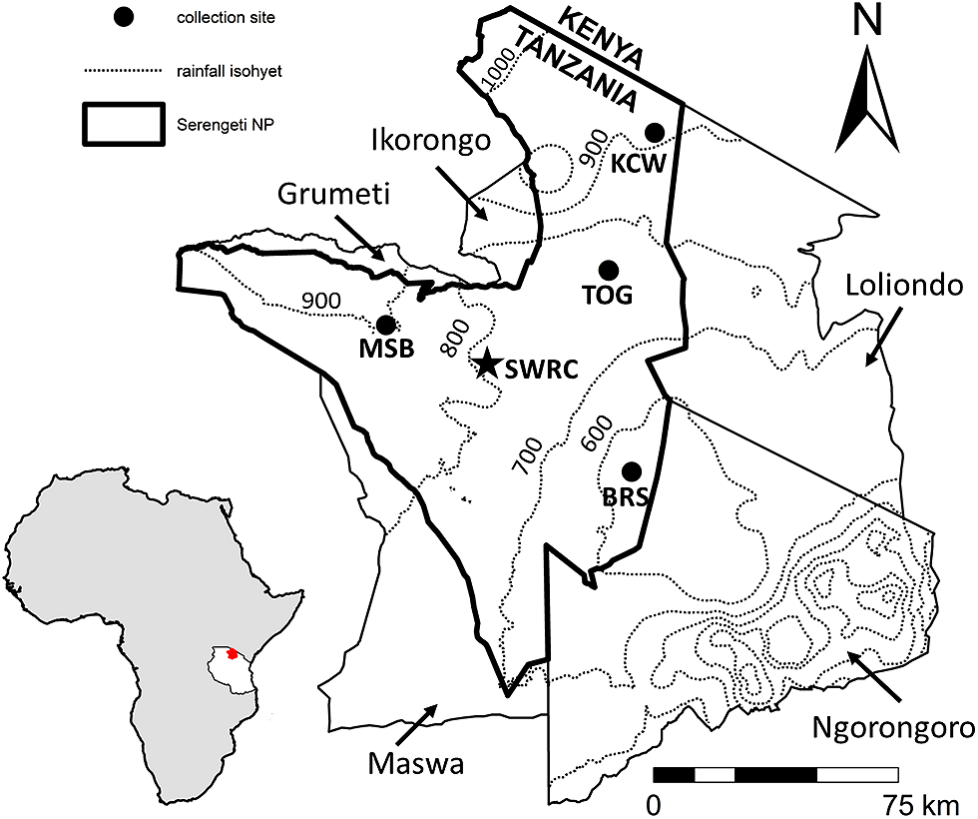
**Map showing the locations of the four collection sites of *Digitaria* and *Themeda* in Serengeti National Park; surrounding protected areas are directly labeled.** Inset of Africa shows the location of this region in northern Tanzania in red. The common garden was constructed at the central Serengeti Wildlife Research Centre (SWRC). Mean annual precipitation is approximately 498 mm/yr at BRS, 676 mm/yr at TOG, 766 mm/yr at KCW, and 891 mm/yr at MSB (values from Anderson et al., 2007).

Our study was conducted in the Serengeti ecosystem in northern Tanzania (2° 19′ 51′′ S, 34° 50′ 0′′ E). Serengeti is characterized by *Acacia*-*Commiphora* dominated savanna vegetation in the north and west of the ecosystem and edaphic grassland on volcanic soils in the Serengeti plains in the southeast (White, 1983). A rain-shadow created by the volcanic highlands to the south and east of Serengeti creates a relatively strong precipitation gradient, in which rainfall decreases from >1100 mm yr-1 in the northwest near the shores of Lake Victoria to ∼600 mm yr-1 in the Serengeti plains at the base of the Ngorongoro crater in the southeast (**Figure 1**). Rainfall is highly seasonal and typically falls in two characteristic phases: the short rains, occurring from November–December, and the long rains, occurring from February–May (Anderson et al., 2007). The soils of the plains are heavily influenced by recent (∼20,000 –1,500 ya) volcanic activity and are highly porous with an underlying calcium carbonate hardpan, resulting in highly saline and alkaline soils with poor moisture retention (Anderson and Talbot, 1965; de Wit, 1978). Interestingly, recent eruptions of nearby volcano Oldoinyo Lengai, which continue to enrich soils in the plains with ash, are of natrocarbonatite origin, meaning that, in addition to being highly enriched in potassium and sodium carbonates, they are extremely depleted of silica (Gittins, 1998; Keller et al., 2010). In contrast, the woodlands to the north are derived from granitic and quartzite parent material, while the western regions of the park are characterized by red clays (Ultisols) and black cotton soils (Vertisols) resulting from alluvial processes associated with Lake Victoria (Jager, 1982).

Serengeti is a “model” grazing ecosystem: massive herds of migratory zebra (*Equus quagga boehmi*) and wildebeest (*Connochaetes taurinus*) exert strong defoliation effects on the grasses that dominate the herbaceous layer. However, the frequency and intensity of herbivory changes along the rainfall gradient, with the most frequent and intense herbivory occurring in the Serengeti plains and decreasing with rainfall. Other significant herbivore species in Serengeti include Thomson’s gazelle (*Eudorcas thomsonii*), which also migrate, and several non-migratory herbivores such as Grant’s gazelle (*Nanger granti*), hartebeest (*Alcelaphus buselaphus*), and topi (*Damaliscus korrigum*). Running counter to the gradient in herbivory is an opposing gradient in fire frequency and intensity created by the tall, highly flammable, bunch grasses in the north and the procumbent, grazing tolerant grasses in the plains to the southeast that rarely burn due to their low aboveground biomass (Anderson et al., 2012). The two most dominant herbaceous species in Serengeti, in terms of both biomass and frequency, are the grasses *T. triandra*, a tall, caespitose bunch grass, and *D. macroblephara*, a short-stature, grazing tolerant lawn grass.

#### Experimental design

In of June 2008, multiple individuals of *T. triandra* and *D. macroblephara*, were collected from four grassland sites spanning the ecosystem’s soil and rainfall gradients and transplanted into a common garden constructed at the Serengeti Wildlife Research Centre (SWRC; **Figure 1**). *D. macroblephara* was collected from Barafu (BRS) in the southeastern plains, *T. triandra* was collected from Klein’s camp (KCW) in the northern corridor, and both species were collected from the Togoro plains (TOG) and Musabi plains (MSB). Grasses were planted in three replicate blocks; each block consisted of a 6 × 9 m fenced area cleared of all vegetation and included six 2 × 2 m equally spaced plots. Three randomly selected individuals of *T. triandra* and *D. macroblephara* (one from each collection site) were planted in each plot. Mean ± SE tiller number of initial *D. macroblephara* individuals was 13.59 ± 0.67, and *T. triandra* transplants had a mean ± SD = 9.08 ± 0.74 tillers. Plots were randomly assigned to one of the six combinations of clipping (two levels: clipped and unclipped) and watering (three levels: high, ambient and low). Water was manipulated by diverting rainfall with five 25 cm strips of clear plastic roof material placed across the plots at ∼6 cm height and slanted slightly to promote water runoff; the treatments were designed to intercept approximately 50% of ambient rainfall. Grasses were planted between the roof strips and at least 45 cm from the nearest grass to provide room for growth and to prevent light competition. To control for any potential effects of the roofing material, all plots were overlaid by the roofing material but holes were drilled in the high and ambient treatments so that no water was diverted and all rainwater reached the plot. Rainfall was collected, and the high water treatment was augmented with an amount that totaled ∼150% of ambient every 2 weeks. Rainfall at SWRC is approximately 700 mm yr^-1^ and the high water treatment was augmented to approximately 1000 mm yr^-1^. Soil moisture at a depth of 12 cm was estimated via time-domain reflectometry monthly in each plot with a FieldScout TDR 100 (Spectrum Inc.; Aurora, IL, USA). In the clipping treatment, plants were defoliated to approximately 50% of their aboveground biomass every 2 months. Clipped biomass was dried, weighed, and summed with harvested biomass to arrive at a final biomass for clipped plants. Unclipped plants were not defoliated throughout the entire duration of the experiment. Plants were grown in the common garden for 10 months, until they were harvested in April 2009. All plants still alive at the harvest were separated into above- and belowground fractions; the aboveground fraction was further separated into leaf and stem. All plant material was dried and weighed in the lab at SWRC; leaf fractions were transported to the US, ground in a Cyclone sample mill (UDY corp.; Fort Collins, CO, USA) and stored until they were analyzed for leaf silica at Wake Forest University.

#### Biogenic silica quantification

Silicon content of leaf tissue was quantified by plasma spectroscopy (ICP-OES) after autoclave-induced digestion (AID) following the methods of Kraska and Breitenbeck (2010; modified from Elliott and Snyder, 1991). Briefly, 100 mg of dried, ground sample was wetted with 1-octanol in a 50 ml vortex tube. Next, 2 mL of 50% H_2_O_2_ and 3.5 mL of 50% NaOH were added. Samples were vortexed several times, until the reaction ceased, and autoclaved loosely capped at 121°C (20 psi) for one hour. Deionized water was added to 50 mL. Samples were brought to acidic pH using concentrated HCl, and diluted 1:10 in deionized water before analyzing by ICP-OES. Si content of samples was calculated by fitting peak intensity at 251 nm to a standard curve (0.1 –10 ppm Si; *r*^2^ ≥ 0.998). The standard curve was validated with a reference material of *Schizachyrium scoparium* (14 g/kg Si). Si values were converted to silica (SiO_2_) content by dividing by a conversion factor of 0.4674, since this more commonly reported value represents biogenic silica (phytolith) content of plant tissue.

#### Statistical analysis

A major goal of our statistical analysis was to understand how defoliation, water addition and their interaction, influenced leaf silica concentration in these two dominant Serengeti grasses. Therefore, we used linear mixed effect (LME) models to test the effects of species, clipping, and water on foliar silica accumulation. The model included species, clipping, and water as fixed factors, while block was included as a random effect to account for the spatial design. Analysis was performed using the lmer function in *lme4* package of R statistical environment version 2.11.1 (Bates et al., 2014; R Development Core Team, 2014), with the original model structure: lmer(SiO2 ∼ species ^∗^ clip ^∗^ water + (1| block)), where “species^∗^clip^∗^water” represents the three-way fixed interaction effect and “(1| block)” represents the random intercepts that are estimated for each block. The function “step” from the R package *lmerTest* was used to simplify the model so that only significant fixed effects remained in the model, and pairwise contrasts for individual treatment effects were subsequently calculated using the “ghlt” command and a Tukey’s test from the R package *multcomp* (Hothorn et al., 2008).

A second model was employed to explore the extent to which collection site could explain variation in foliar silica content for both species. Because we did not have sufficient replication of individual grasses at the site level, we could not explore the effects of site in a full model crossed with our other treatments. Consequently, we used a reduced statistical model to explore the main effects of site on leaf silica for each individual species averaged over all levels of water and defoliation. The model we employed for each species separately was: lmer(SiO2 ∼ site + (1| block)). Model simplification was again conducted via the “step” function in R.

Finally, soil moisture was statistically compared across treatments using a model with water as a fixed effect and time and block as random effects using the lmer function in *lme4* package of R as described above; the “ghlt” command from the R package *multcomp* was used to conduct a Tukey’s *post hoc* comparison of means.

### LITERATURE REVIEW

In order to identify general trends in plant response to defoliation, we conducted an extensive search of primary literature sources to identify studies which provided data on silica content of Poales under both control and defoliation treatments. We used Google Scholar and Web of Science to identify appropriate primary research articles by using combinations of search terms such as “clipping,” “grazing,” “defoliation,” “silica,” and “phytolith.” Within the order Poales, defoliation studies were only identified for grasses, so the final literature review is limited to Poaceae. We restricted our search to studies containing species-specific silica values in order to avoid the potential confounding effects of differing community composition on bulk, plot level silica content. These studies included laboratory studies and field studies in which grazing was experimentally prevented by herbivore exclosures. Grazing studies encompassed insect, small mammal, and large mammal herbivory, and the intensity of defoliation varied within and among studies (see Discussion). Species-specific Si values were converted to SiO_2_ when necessary, assigned a unique identifier, and plotted as the log ratio normalized difference between defoliated and non-defoliated plants at the species level. Studies were then organized by defoliation method and grazer type and assigned to a general defoliation response category to facilitate interpretation of the silica response. Those cases with a ≥ 20% increase in silica following defoliation were assigned a “+,” those which decreased ≥20% were assigned a “-,” and those exhibiting less than a 20% relative change in silica content were assigned a “0.”

## RESULTS

### SERENGETI COMMON GARDEN

Mean ± SE soil moisture values were 11.1 ± 1.6% for the high, 9.8 ± 1.5% for the ambient and 9.7 ± 1.5% for the low watering treatment. *Post hoc* statistical comparisons with a Tukey’s test demonstrated that the low and ambient water treatments were not statistically different from one another (difference = -0.1, *z* = -0.26, *p* = 0.96), but that the high water treatment was statistically greater than the low (difference = 1.4, *z* = -2.9, *p* = 0.007) and ambient (difference = 1.3, *z* = 2.73, *p* = 0.01) treatments. Therefore, the ambient and low water treatments are combined for the remainder of the manuscript (referred to as “ambient” from here onward) and compared to the high water treatment in all analyses of watering effects.

**FIGURE 2:**
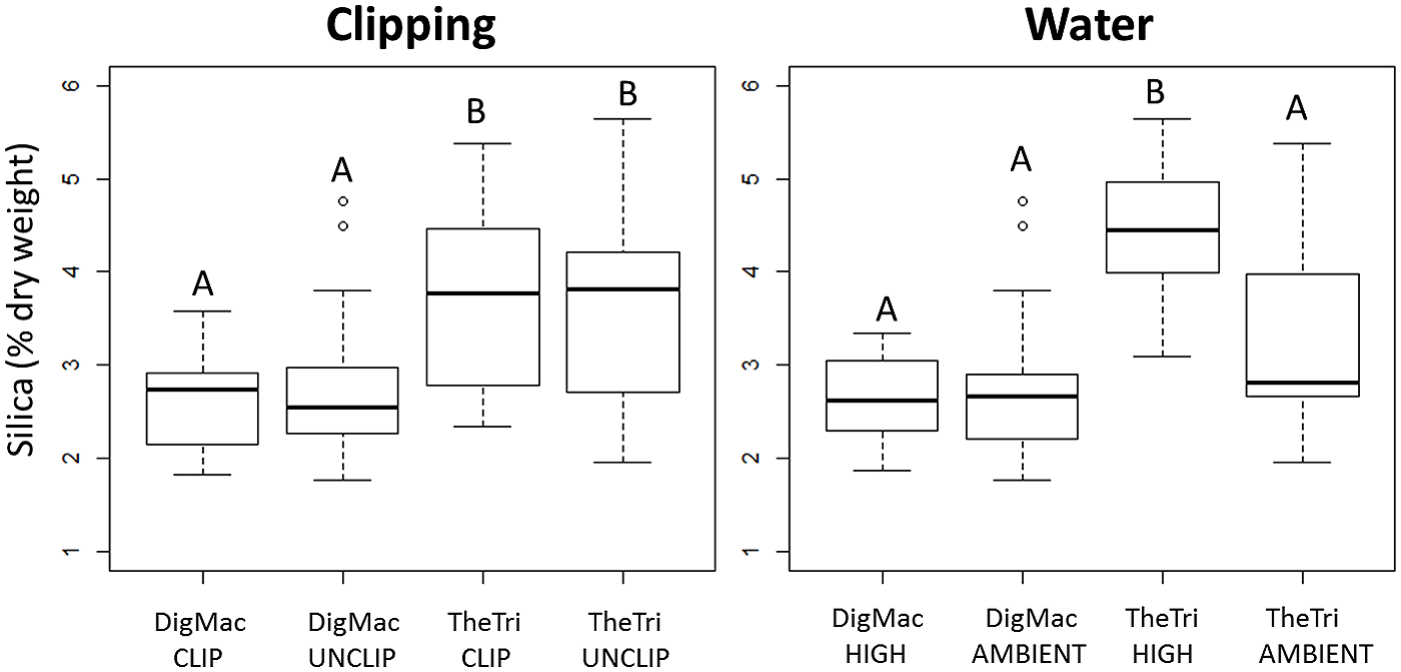
**Silica content of *Digitaria macroblephara* (DigMac) and *Themeda triandra* (TheTri) leaves as affected by defoliation and watering treatments.** Defoliated individuals were clipped to 50% of maximum height bi-monthly a total of four times. High water treatment individuals received approximately 150% of ambient rainfall, and ambient treatment represents low and ambient water groups pooled (see Materials and Methods). Groups which differ significantly are indicated by a letter change.

Both the bunch-grass *T. triandra* and lawn-grass *D. macroblephara* were relatively high Si-accumulators: *T. triandra* had a mean ± SE foliar SiO_2_ content of 3.7 ± 0.25 % dw (*n* = 20), while *D. macroblephara* had a mean ± SE SiO_2_ of 2.7 ± 0.10 % (*n* = 41). Neither species showed a significant response to clipping as a main effect (*p* = 0.976) or as an interaction effect when clipping was crossed with watering level (*p* = 0.453; **Figure 2**). However, there was a significant species by watering interaction effect (*p*<0.05, Supplementary Table S1) indicating that leaf silica concentrations of the two species differed in response to the watering treatment. This result arose because individuals of *T. triandra* that were watered had a higher leaf silica concentration (4.4 ± 0.3%) compared to those *T. triandra* plants that were maintained at ambient soil moisture levels (3.3 ± 0.3%). In contrast, no significant change in SiO_2_ content was observed between watering treatments for *D. macroblephara* (**Figure 2**).

**FIGURE 3:**
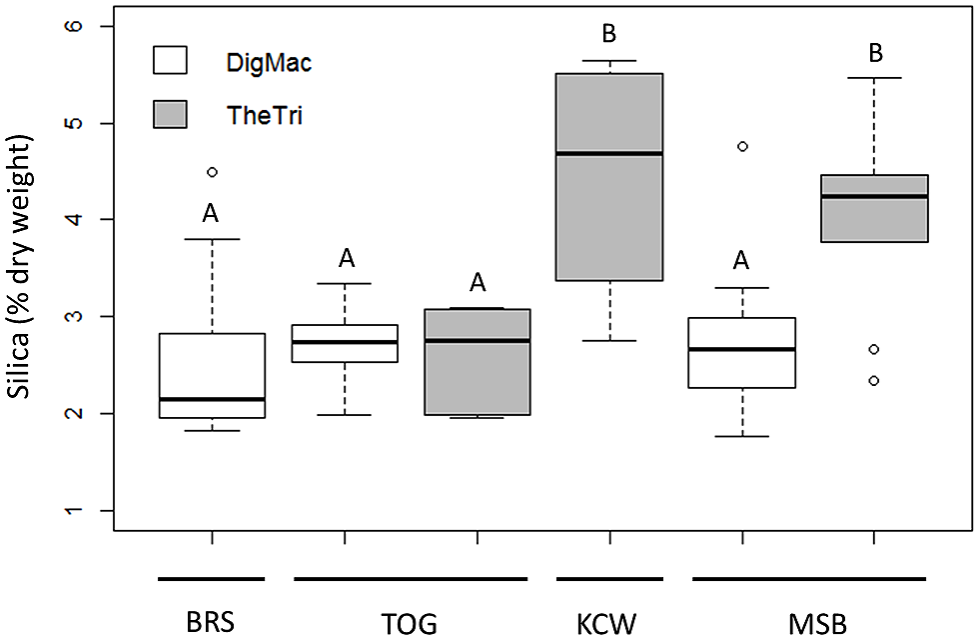
**Foliar silica content of each species in relation to plant collection sites.** Both species were collected at the intermediate sites, MSB and TOG. *Themeda* (TheTri) does not occur at the shortgrass plains site BRS, and *Digitaria* (DigMac) does not occur at KCW in the northern woodlands. Sites are arranged in order of increasing mean annual precipitation: BRS = 498 mm/yr, TOG = 676 mm/yr, KCW = 766 mm/yr, MSB = 891 mm/yr.

In the second analysis, in which the statistical model included the main effects of plant collection site for each species separately, *D. macroblephara* exhibited considerable within-site variability and consequently no statistically significant differences across sites. In contrast, the SiO_2_ content of *T. triandra* plants varied significantly among sites (**Figure 3**; see **Figure 1** for sites): Silica was lowest for plants collected from TOG (mean ± SE = 2.67 ± 0.63%), intermediate for plants from MSB (mean ± SE = 3.97 ± 0.55%), and highest for plants from KCW (mean ± SE = 4.18 ± 0.53%). Independent contrasts indicated that grasses from the low rainfall site TOG accumulated significantly less silica than those collected from MSB (difference = -1.30, *z* = -2.822, *p* = 0.005) and KCW (difference = -1.51, *z* = -2.408, *p* = 0.016).

### LITERATURE REVIEW

**FIGURE 4:**
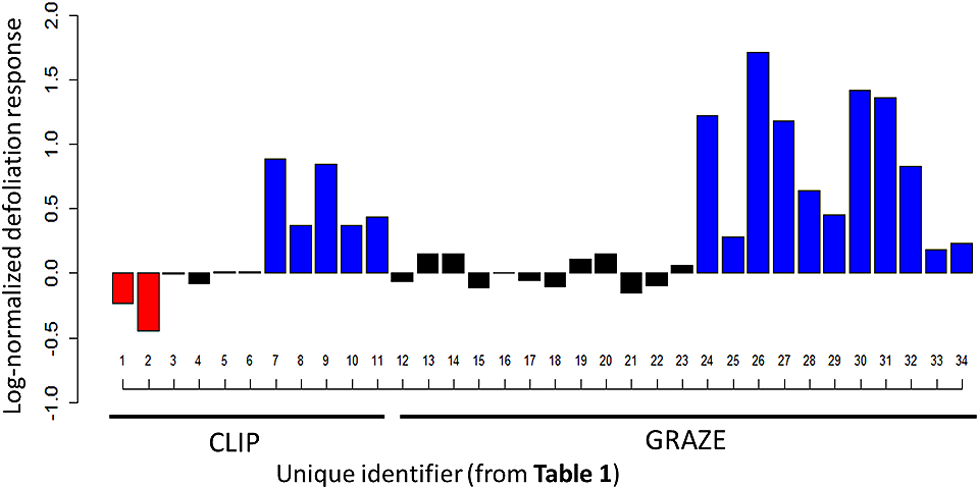
**Log-normal change in foliar silica content of defoliated grasses identified in the literature review.** Blue bars indicate a ≥ 20% increase in silica following defoliation, red bars indicate a ≥ 20% decrease, and black bars indicate <20% change in either direction. Individual responses are arranged according to unique identifiers from Supplemenatry Table S2; Unique identifiers 1–11 represent clipping studies, and 12–34 represent grazing studies.

We identified 11 studies conducted from 1974-present which recorded species-specific silica induction under control and defoliation treatments. These studies represented 34 “cases” of potential silica induction for 15 different grass species (Supplementary Table S2). Due to the limited number of studies conducted and lack of species-specific replication, we could not employ meta-analytical statistical approaches; instead, general responses were considered and interpreted. Eleven of fifteen species surveyed are C3, and all are perennials, except for *Poa annua*. Silica content of surveyed species ranged from <0.5% dry weight (non-defoliated *Anthoxanthum odoratum*) to >7% (grazed *Pascopyrum smithii*, *Deschampsia cespitosa,* and *Eustachys paspaloides*). To avoid pseudoreplication, we reported silica values for seasonal or site-level maxima for the three studies which reported multiple species-specific responses to the same defoliation method (Brizuela and Detling, 1986; Cid et al., 1990; Banuelos and Obeso, 2000). The majority of studies surveyed suggest that plants are able to respond to defoliation by altering their silica uptake, and indeed we observed an overall trend of silica induction following defoliation (**Figure 4**). Despite this general trend, several studies found no significant change in silica content of defoliated grasses, defined here as less than a 20% change in foliar silica content in either direction. The literature review revealed substantial variation in the silica responses of the different species studied. For example, the C3 grass *Festuca* increased silica content more than 350% in response to herbivory by voles (Massey et al., 2007), while silica content of *Pascopyrum* decreased by approximately 50% following clipping (Cid et al., 1990). In general, the magnitude of induced silica uptake was greater under natural defoliation (i.e., grazing) than for mechanical clipping. Further, the magnitude of silica increase when defoliation stimulated uptake much greater than the decrease in silica levels observed in the two studies in which clipping triggered down regulation of silica levels.

## DISCUSSION

### VARIATION IN SILICA BETWEEN *Themeda* AND *Digitaria*

Both species documented in this study were within the typical silica range for dryland grasses, but *Themeda* had significantly greater silica content than *Digitaria.* These two species have different suites of anatomical and physiological traits and may be considered distinct graminoid functional types (Coughenour, 1985; Diaz and Cabido, 1997). Lawn grasses, such as *Digitaria*, exhibit clonal, stoloniferous growth; their prostrate growth form allows them to reduce tissue loss to herbivores while quickly spreading horizontally to form lawns under high soil fertility (Cromsigt and Olff, 2008). In contrast, the higher lignin content of tall, dense, and slow-growing bunch grass species, like *Themeda*, makes them less palatable to herbivores than lawn grasses (Sinclair and Norton-Griffiths, 1979). Thus, as a consequence of their different life history strategies, it is not surprising that these two species differ in the degree to which silica accumulates in their leaves.

As a documented growth promoter (McNaughton et al., 1985; Isa et al., 2010) and metabolically “cheap” structural substitute for carbon-based compounds such as lignin (Raven, 1983; Cooke and Leishman, 2011a), silica deposition may provide an alternative mechanism for accelerated growth which would prove especially beneficial for bunch grasses. The increased growth rates that result from silica accumulation may substantially improve light interception of slow-growing species which primarily compete for sunlight (Ando et al., 2002). This idea is further supported by a positive correlation between leaf length and silica content observed in *Spartina* (Querné et al., 2012), again supporting the idea that silica allows improved growth without requiring significant carbon investment (Cooke and Leishman, 2012). Further, slow-growing bunch grasses display greater leaf mass per area, LMA, (Fynn et al., 2011) and leaf dry matter content, LDMC, (Anderson et al., 2013) than fast-growing lawn species, indicating a greater relative investment in dry matter. The worldwide leaf economic spectrum (Wright et al., 2004) predicts a strong positive correlation between dry matter investment and leaf longevity, suggesting that, based on their higher LMA, bunch-grass species likely also have leaves of greater longevity than lawn-grass species. Thus, it may be beneficial for relatively long-lived bunch grasses to invest in immobile phytoliths (Endara and Coley, 2011) which allow for rapid augmentation of cell structure of C-limited species under intense light competition. On the other hand, recent research revealed a negative correlation between leaf lifespan and Si concentration when considering a broad range of plant functional types (Cooke and Leishman, 2011b). The reasons for differential responses to defoliation and high interspecific variation in this qualitatively ubiquitous defense within Poaceae remain unclear. Here we focused on two species representing extremes of each of the two grass growth forms (bunch, lawn); greater sampling from along the gradient of short to tall grasses is needed to better understand the influence of these contrasting physiological forms on silica accumulation.

### DEFOLIATION RESPONSE

Contrary to our initial predictions, clipping did not result in silica induction for either the lawn or bunch grass species, with nearly identical mean silica content observed for defoliated and non-defoliated individuals. There are several possible explanations for the lack of silica induction observed in our Serengeti common garden study. First, the frequency and/or intensity of defoliation may not have been great enough to elicit a response. For example, in the Serengeti plains, more than 1 million migratory ungulates are present in dense herds during the wet season, and localized grazing “hotspots” support dense spatially and temporally stable grazer communities that inflict especially frequent and intense herbivory on plant communities (Holdo et al., 2009; Anderson et al., 2010). Our literature review further corroborates this notion. For example, a laboratory study by Massey et al. (2007) revealed that a single defoliation event did not induce silica uptake, while repeated defoliation by herbivores (16 events total) significantly increased silica content of ryegrass and fescue, revealing the importance of both frequency and duration of defoliation. Extent of tissue removal (5% vs. 25%) is also proven to affect both induction and relaxation of silica response, demonstrating a threshold effect required for induction (Reynolds et al., 2012). In our study, grasses were defoliated on a bi-monthly basis, four times total. For at least certain areas of Serengeti, this may be lower than levels of natural grazing intensity due to Serengeti’s rich herbivore fauna.

In addition, manual defoliation (i.e., clipping) likely does not elicit the same response as natural grazing under laboratory or field conditions, a well-known effect with respect to induced defenses. Our literature review revealed that clipping and grazing both resulted in silica induction in ∼50% of the studies (5 of 11 clipping cases, 12 of 23 grazing cases), but the magnitude of this response was much greater under grazing defoliation, as illustrated in **Figure 4**. Among grazing studies, silica induction was observed in response to insect, small mammal, and large mammal grazing. We propose that clipping may not be sufficient to represent both the direct and indirect effects of grazing on silica dynamics in grasses; this may be especially true for grass species growing in the Serengeti plains, which have a long co-evolutionary history with herbivores. Large-bodied grazers compact soils, resulting in increased bulk density (e.g., Bakker et al., 2004; Holdo and Mack, 2014; Veldhuis et al., 2014) and matric water potential and, thus, alter the ability of roots to absorb water. While manual defoliation studies yield information about certain physiological responses, the literature review presented here suggests that this approach may not properly mimic the complex physiological responses associated with natural grazing, some of which may result from indirect effects associated with modification of soil water (see below). In summary, plants perceive herbivore effects through a suite of signals, some of which, such as changes in the abiotic environment, are not elicited directly by the removal of tissues.

### WATER AND COLLECTION SITE EFFECTS

*Themeda* individuals in the high water treatment accumulated significantly greater foliar silica than those in the ambient water group (**Figure 2**), but this trend was not observed for *Digitaria*. This suggests either differential silica uptake mechanisms of the two species or different generalized physiological responses to drought which indirectly influence silica accumulation. Since *Themeda* responded to changes in water availability, we hypothesize that active uptake mechanisms are present in this species which allow it to increase uptake of Si(OH)_4_ from the soil solution when water is abundant. If true, this then raises the question, why would *Digitaria* not also increase uptake of a ubiquitously beneficial element under similar conditions? One possible explanation is that the different life histories of these two species, coupled with the proposed costs of silica accumulation (Cooke and Leishman, 2012) have resulted in selection for differential silica uptake mechanisms, in which tall, bunch species but not short, lawn species, have incorporated active Si uptake mechanisms such as the use of specialized energy-dependent transport molecules. *Digitaria* has highly flexible leaves which are capable of curling under drought conditions to prevent transpiration; this drought tolerance mechanism would likely be hindered due to the loss of bulliform cell function associated with leaf tissue silicification (Honaine and Osterrieth, 2011). Conversely, the benefit of improved drought tolerance associated with silica accumulation (Gao et al., 2006; Eneji et al., 2008) is likely of greater relative importance for bunch grass species, such as *Themeda*, which experience intense light competition and a significant seasonal water deficit. Increased silica uptake under high water availability may serve to buffer bunch grasses against future drought events while simultaneously producing an erect canopy structure (see Isa et al., 2010) for light competition and offsetting carbon costs. Under this proposed hypothesis, that bunch grasses benefit more than lawn grasses due to more intense light competition, any anti-herbivore benefits are secondary to the direct adaptive significance of silica accumulation, which is related to water and/or light limitation

Consistent with the observed effects of watering treatments, the silica differences among collection sites also indicate that water availability may be an important determinant of grass foliar silica content. *Themeda* individuals collected from high rainfall sites KCW and MSB, where light competition is expected to be more intense, exhibited significantly greater silica content than individuals collected from the low rainfall site TOG, where light competition is expected to be less intense. In contrast, the leaf silica content of *Digitaria* individuals was not influenced by collection site (**Figure 3**). This observation reinforces the notion that water strongly influences foliar silica content of grasses and implies a pattern of increasing silica content of tall bunch grass species, like *Themeda*, with increasing distance from the plains and increasing light competition.

Other possible explanations exist for the observed site-level variation in silica content of *T. triandra*. Numerous fertilization studies have indicated that plant silica content is strongly correlated with availability of Si(OH)_4_ in the soil medium (Fox et al., 1969; Jones and Handreck, 1969; Van der Vorm, 1980; Gali and Smith, 1992), and more recently, ecological studies have demonstrated a similar relationship between soils and plants in natural systems. Biogenic silica content of grasses in a South African savanna was higher for plants collected from basaltic soils than for those collected from granitic soils, reflecting differences in weatherability and dissolved silica (DSi) content of these contrasting parent materials (Melzer et al., 2011). Cooke and Leishman (2012) compared foliar silica content of plant communities from Hawkesbury sandstone and a nearby diatreme forest with nearly three times as much plant available soil Si; though not statistically significant, they observed a general trend of higher foliar silica content for plants from the diatreme site, including a fivefold difference in leaf silica observed for the sedge *Schoenus melanostachys*. The unique nature of the silica-depleted carbonatite ash that enriches the Serengeti plains may contribute to a gradient in available soil Si, in which available Si increases toward the northern and western corridors of the park. However, more work is needed to document how soil Si availability varies within SNP and how it may interact with gradients in soil moisture, pH, and other important soil nutrients known to vary across the landscape (de Wit, 1978; Jager, 1982). Finally, we caution that, due to geographic separation, plant genotype should also vary among sites (Soininen et al., 2013), and we cannot rule out genotypic variation as an important influence on silica accumulation patterns.

### ECOLOGICAL AND EVOLUTIONARY IMPLICATIONS

The results of our literature review suggest that induced silica response to defoliation is dynamic and dependent upon interactions with additional biotic and abiotic factors. For example, defoliation by burning resulted in more than a 1/3 decrease in silica content of *Aristida* grass (Dufek et al., 2014). Moreover, seasonal (Georgiadis and McNaughton, 1988, 1990; Carey and Fulweiler, 2013; Struyf et al., 2005) and inter-annual (Morton and Jutras, 1974; Pahkala and Pihala, 2000) variations in field measurements of grass silica content indicate that leaf tissue silicification is highly plastic. While seasonal fluctuations in grass silica content are well-documented, it remains unclear whether dynamic silica values measured in the field occur in response to changes in grazing intensity, temperature, precipitation, or, most likely, a complex interaction among these stochastic variables.

Silica accumulation is considered a central axis of grass–grazer coevolution throughout the literature, often cited as a driving force behind the evolution of hypsodonty and the coupled taxonomic radiation of grasses and grazers (Mendoza and Palmqvist, 2008; Hummel et al., 2011). The evolution of large-bodied mammalian grazers is thought to have selected for specialized traits of the Poaceae such as meristematic growth from a well-protected crown, rapid growth rate, and high levels of Si accumulation (McNaughton, 1979; Coughenour, 1985). Likewise, these plant traits are thought to have influenced herbivore dentition, digestion, and behavior (McNaughton, 1984; Massey and Hartley, 2006; Erickson et al., 2012). Our study and literature review, however, suggest that the evolutionary significance of silica accumulation may be a remnant of tradeoffs associated with the leaf economic spectrum that resulted in the evolution of two contrasting grass functional types. Silica accumulation may have first served to improve drought tolerance with herbivore deterrence as a secondary role. Fossil data support this notion: grass-dominated habitats preceded the appearance of North American and Mediterranean grazing specialists (Strömberg, 2006; Strömberg et al., 2007), and an investigation of the Gran Barranca fossil record indicates that open grass habitats were not necessary pre-conditions for favoring early hypsodont mammals in Earth’s earliest grass-dominated systems (Strömberg et al., 2013).

## Conflict of Interest Statement

The authors declare that the research was conducted in the absence of any commercial or financial relationships that could be construed as a potential conflict of interest.

## SUPPLEMENTARY MATERIAL

Table S1 **Results from linear mixed model in R testing for interactions between species and water on foliar silica accumulation.** Block was included as a random effect. *P* values in bold indicate significance at α = 0.05.

Table S2 **Summary of studies included in literature review of induced silica response of Poaceae; colors in the “Defoliation Response” column correspond with Figure 4.** In the *Defoliation Response* column, “0” indicates that a species exhibited less than a 20% relative change in foliar silica content in response to defoliation, while “**+**” indicates a ≥ 20% increase in silica, and a “**-**” indicates a ≥ 20% decrease in silica.

## References

[B1] Agarie S. Hanaoka N. Ueno O. Miyazaki A. Kubota F. Agata W. (1998). Effects of silicon on tolerance to water deficit and heat stress in rice plants, monitored by electrolyte leakage. *Plant Prod. Sci.* 1 96–103. 10.1626/pps.1.96

[B2] Agrawal A. A. Rutter M. T. (1998). Dynamic anti-herbivore defense in ant-plants: the role of induced responses. *Oikos* 83 227–236. 10.2307/3546834

[B3] Anderson G. D. Talbot L. M. (1965). Soil factors affecting the distribution of the grassland types and their utilization by wild animals on the serengeti plains, tanganyika. *J. Ecol.* 53 33–56. 10.2307/2257564

[B4] Anderson T. M. Hopcraft J. G. Eby S. Ritchie M. Grace J. B. Olff H. (2010). Landscape-scale analyses suggest both nutrient and antipredator advantages to serengeti herbivore hotspots. *Ecology* 91 1519–1529. 10.1890/09-0739.1 20503883

[B5] Anderson T. M. Kumordzi B. B. Fokkema W. Fox H. V. Olff H. (2013). Distinct physiological responses underlie defoliation tolerance in african lawn and bunch grasses. *Int. J. Plant Sci.* 174 769–778. 10.1086/670237

[B6] Anderson T. M. Ritchie M. E. McNaughton S. J. (2007). Rainfall and soils modify plant community response to grazing in serengeti national park. *Ecology* 88 1191–1201. 10.1890/06-0399 17536405

[B7] Anderson T. M. Schütz M. Risch A. C. (2012). Seed germination cues and the importance of the soil seed bank across an environmental gradient in the Serengeti. *Oikos* 121 306–312. 10.1111/j.1600-0706.2011.19803.x

[B8] Ando H. Kakuda K. Fujii H. Suzuki K. Ajiki T. (2002). Growth and canopy structure of rice plants grown under field conditions as affected by Si application growth and canopy structure of rice plants grown under field conditions as affected by Si application. *Soil Sci. Plant Nutr.* 48 429–432. 10.1080/00380768.2002.10409221

[B9] Arimura G. Ozawa R. Shimoda T. Nishioka T. Boland W. Takabayashi J. (2000). Herbivory-induced volatiles elicit defence genes in lima bean leaves. *Nature* 406 512–515. 10.1038/35020072 10952311

[B10] Bakker E. S. Olff H. Boekhoff M. Gleichman J. M. Berendse F. (2004). Impact of herbivores on nitrogen cycling: contrasting effects of small and large species. *Oecologia* 138 91–101. 10.1007/s00442-003-1402-5 14566555

[B11] Banuelos M. J. Obeso J. R. (2000). Effect of grazing history, experimental defoliation, and genotype on patterns of silicification in agrostis tenuis sibth. *Ecoscience* 7 45–50

[B12] Bates D. Maechler M. Bolker B. Walker S. (2014). *_lme4: Linear Mixed-Effects Models using Eigen and S4_*. R Package Version 1.1-7. Available at: https://github.com/lme4/lme4/; http://lme4.r-forge.r-project.org/

[B13] Brizuela M. A. Detling J. K. (1986). Silicon concentration of grasses growing in sites with different grazing histories. *Ecology* 67 1098–1101. 10.2307/1939834

[B14] Carey J. C. Fulweiler R. W. (2013). Nitrogen enrichment increases net silica accumulation in a temperate salt marsh. *Limnol. Oceanogr.* 58 99–111. 10.4319/lo.2013.58.1.0099

[B15] Cid M. S. Detling J. K. Whicker A. D. Brizuela M. A. (1990). Silicon uptake and distribution in agropyron smithii as related to grazing history and defoliation. *J. Range Manag.* 43 344–346. 10.2307/3898929

[B16] Cooke J. Leishman M. R. (2011a). Is plant ecology more siliceous than we realise? *Trends Plant Sci.* 16 61–68. 10.1016/j.tplants.2010.10.003 21087891

[B17] Cooke J. Leishman M. R. (2011b). Silicon concentration and leaf longevity: is silicon a player in the leaf dry mass spectrum? *Funct. Ecol.* 25 1181–1188. 10.1111/j.1365-2435.2011.01880.x

[B18] Cooke J. Leishman M. R. (2012). Tradeoffs between foliar silicon and carbon-based defences: evidence from vegetation communities of contrasting soil types. *Oikos* 121 2052–2060. 10.1111/j.1600-0706.2012.20057.x

[B19] Cotterill J. V. Watkins R. W. Brennon C. B. Cowan D. P. (2007). Boosting silica levels in wheat leaves reduces grazing by rabbits. *Pest Manag. Sci.* 63 247–253 17177170 10.1002/ps.1302

[B20] Coughenour M. B. (1985). Graminoid responses to grazing by large herbivores: adaptations, exaptations, and interacting processes. *Ann. Mo. Bot. Gar.* 72 852–863. 10.2307/2399227

[B21] Cromsigt J. P. G. M. Olff H. (2008). Dynamics of grazing lawn formation: an experimental test of the role of scale-dependent processes. *Oikos* 117 1444–1452. 10.1111/j.2008.0030-1299.16651.x

[B22] de Wit H. A. (1978). *Soils and Grassland Types of the Serengeti Plain Tanzania.* Ph.D. dissertation, Agricultural University, Wageningen

[B23] Diaz S. Cabido M. (1997). Plant functional types and ecosystem function in relation to global change. *J. Veg. Sci.* 8 463–474. 10.1111/j.1654-1103.1997.tb00842.x

[B24] Dufek N. A. Vermeire L. T. Waterman R. C. Ganguli A. C. (2014). t fire and nitrogen addition increase forage quality of aristida purpurea. *Rang. Ecol. Manag.* 67 298–306. 10.2111/REM-D-13-00057.1

[B25] Elliott C. L. Snyder G. H. (1991). Autoclave-induced digestion for the colorimetric determination of silicon in rice straw. *J. Agric. Food Chem.* 39 1118–1119. 10.1021/jf00006a024

[B26] Endara M. Coley P. D. (2011). The resource availability hypothesis revisited: a meta-analysis. *Funct. Ecol.* 25 389–398. 10.1111/j.1365-2435.2010.01803.x

[B27] Eneji A. E. Inanaga S. Muranaka S. Li J. Hattori T. An P. (2008). Growth and nutrient use in four grasses under drought stress as mediated by silicon fertilizers. *J. Plant Nutr.* 31 355–365. 10.1080/01904160801894913

[B28] Epstein E. (1999). Silicon. *Annu. Rev. Plant Physiol. Plant Mol. Biol.* 50 641–664. 10.1146/annurev.arplant.50.1.641 15012222

[B29] Erickson G. M. Krick B. A. Hamilton M. Bourne G. R. Norell M. A. Lilleodden E. (2012). Complex dental structure and wear biomechanics in hadrosaurid dinosaurs. *Science* 338 98–101. 10.1126/science.1224495 23042891

[B30] Fauteux F. Chain F. Belzile F. Menzies J. G. Bélanger R. R. (2006). The protective role of silicon in the arabidopsis–powdery mildew pathosystem. *Proc. Natl. Acad. Sci. U.S.A.* 103 17554–17559 10.1073/pnas.0606330103 17082308 PMC1859967

[B31] Feeny P. (1976). “Plant apparency and chemical defense,” in *Biochemical Interaction Between Plants and Insects* ed. Wallace J. W. (New York: Plenum Press), 1–40. 10.1007/978-1-4684-2646-5_1

[B32] Fox R. L. Silva J. A. Plucknett D. L. Teranishi D. Y. (1969). Soluble and total silicon in sugar cane. *Plant Soil* 30 81–92. 10.1007/BF01885263

[B33] Fynn R. Morris C. Ward D. Kirkman K. (2011). Trait-environment relations for dominant grasses in south african mesic grassland support a general leaf economic model. *J. Veg. Sci.* 22 528–540. 10.1111/j.1654-1103.2011.01268.x

[B34] Gali H. U. Smith C. C. (1992). Effect of silicon supply on growth, fertility, and mineral composition of an annual brome, bromus secalinus L. *(Gramineae). Am. J. Bot.* 79 1259–1263. 10.2307/2445053

[B35] Gali-Muhtasib H. U. Smith C. C. Higgins J. J. (1992). The effect of silica in grasses on the feeding behavior of the prairie vole, microtus ochrogaster author. *Ecology* 73 1724–1729. 10.2307/1940024

[B36] Galvez L. Clark R. B. Gourley L. M. Maranville J. W. (1987). Silicon interactions with manganese and aluminum toxicity in sorghum. *J. Plant Nutr.* 10 1139–1147. 10.1080/01904168709363642

[B37] Gao X. Zou C. Wang L. Zhang F. (2006). Silicon decreases transpiration rate and conductance from stomata of maize plants. *J. Plant Nutr.* 29 1637–1647. 10.1080/01904160600851494

[B38] Georgiadis N. J. McNaughton S. J. (1988). Interactions between grazers and a cyanogenic grass, cynodon plectostachyus. *Oikos* 51 343–350. 10.2307/3565316

[B39] Georgiadis N. J. McNaughton S. J. (1990). Elemental and fibre contents of savanna grasses: variation with grazing, soil type, season, and species. *J. Appl. Ecol.* 27 623–634. 10.2307/2404307

[B40] Gibson D. J. (2009). *Grasses and Grassland Ecology.* New York, NY: Oxford University Press

[B41] Gittins J. (1998). Differentiation of natrocarbonatite magma at oldoinyo lengai volcano, tanzania. *Mineral. Mag.* 62 759–768. 10.1180/002646198548142

[B42] Heine G. Tikum G. Horst W. J. (2007). The effect of silicon on the infection by and spread of pythium aphanidermatum in single roots of tomato and bitter gourd. *J. Exp. Bot.* 58 569–577. 10.1093/jxb/erl232 17158106

[B43] Hodson M. J. White P. J. Mead A. Broadley M. R. (2005). Phylogenetic variation in the silicon composition of plants. *Ann. Bot.* 96 1027–1046. 10.1093/aob/mci255 16176944 PMC4247092

[B44] Holdo R. M. Holt R. D. Fryxell J. M. (2009). Opposing rainfall and plant nutritional gradients best explain the wildebeest migration in the serengeti. *Am. Nat.* 173 431–445. 10.1086/597229 19243258

[B45] Holdo R. M. Mack M. C. (2014). Functional attributes of savanna soils: contrasting effects of tree canopies and herbivores on bulk density, nutrients and moisture dynamics. *J. Ecol.* 102 1171–1182. 10.1111/1365-2745.12290

[B46] Honaine M. F. Osterrieth M. L. (2011). Silicification of the adaxial epidermis of leaves of a panicoid grass in relation to leaf position and section and environmental conditions. *Plant Biol.* 14 596–604. 10.1111/j.1438-8677.2011.00530.x 22188340

[B47] Hothorn T. Bretz F. Westfall P. (2008). Simultaneous inference in general parametric models. *Biom. J.* 50 346–363. 10.1002/bimj.200810425 18481363

[B48] Hummel J. Findeisen E. Südekum K. Ruf I. Kaiser T. M. Bucher M. (2011). Another one bites the dust: faecal silica levels in large herbivores correlate with high-crowned teeth. *Proc. R. Soc. B Biol. Sci.* 278 1742–1747. 10.1098/rspb.2010.1939 PMC3081769 21068036

[B49] Isa M. Bai S. Yokoyama T. Feng Ma J. Ishibashi Y. Yuasa T. (2010). Silicon enhances growth independent of silica deposition in a low-silica rice mutant, lsi1. *Plant Soil* 331 361–375. 10.1007/s11104-009-0258-9

[B50] Jager T. J. (1982). *Soils of the Serengeti Woodlands,* Wageningen

[B51] Jones L. H. P. Handreck K. A. (1969). Uptake of silica by trifolium incarnatum in relation to the concentration in the external solution and to transpiration. *Plant Soil* 1 71–80. 10.1007/BF01885262

[B52] Keller J. Klaudius J. Kervyn M. Ernst G. G. J. Mattsson H. B. (2010). Fundamental changes in the activity of the natrocarbonatite volcano oldoinyo lengai, tanzania. *Bull. Volcanol.* 72 893–912. 10.1007/s00445-010-0371-x

[B53] Kraska J. E. Breitenbeck G. A. (2010). Simple, robust method for quantifying silicon in plant tissue. *Commun. Soil Sci. Plant Anal.* 41 2075–2085. 10.1080/00103624.2010.498537

[B54] Ma J. F. Tamai K. Yamaji N. Mitani N. Konishi S. Katsuhara M. (2006). A silicon transporter in rice. *Nature* 440 688–691. 10.1038/nature04590 16572174

[B55] Ma J. F. Yamaji N. (2006). Silicon uptake and accumulation in higher plants. *Trends Plant Sci.* 11 392–397. 10.1016/j.tplants.2006.06.007 16839801

[B56] Ma J. F. Yamaji N. Mitani N. Tamai K. Konishi S. Fujiwara T. (2007). An eﬄux transporter of silicon in rice. *Nature* 448 209–212. 10.1038/nature05964 17625566

[B57] Massey F. P. Ennos A. R. Hartley S. E. (2007). Herbivore specific induction of silica-based plant defences. *Oecologia* 152 677–683. 10.1007/s00442-007-0703-5 17375331

[B58] Massey F. P. Hartley S. E. (2006). Experimental demonstration of the antiherbivore effects of silica in grasses: impacts on foliage digestibility and vole growth rates. *Proc. R. Soc. B Biol. Sci.* 273 2299–2304. 10.1098/rspb.2006.3586 PMC1636089 16928631

[B59] Massey F. P. Hartley S. E. (2009). Physical defences wear you down: progressive and irreversible impacts of silica on insect herbivores. *J. Anim. Ecol.* 78 281–291. 10.1111/j.1365-2656.2008.01472.x 18771503

[B60] Mauricio R. Rausher M. D. Burdick D. S. (1997). Variation in the defense strategies of plants: are resistance and tolerance mutually exclusive? *Ecology* 78 1301–1311. 10.1890/0012-9658(1997)078[1301:VITDSO]2.0.CO;2

[B61] McNaughton S. J. (1979). Grazing as an optimization process: grass-ungulate relationships in the serengeti. *Am. Nat.* 113 691–703. 10.1086/283426

[B62] McNaughton S. J. (1984). Grazing lawns: animals in herds, plant form, and coevolution. *Am. Nat.* 124 863–886. 10.1086/284321

[B63] McNaughton S. J. Tarrants J. L. (1983). Grass leaf silicification: natural selection for an inducible defense against herbivores. *Proc. Natl. Acad. Sci. U.S.A.* 80 790–791. 10.1073/pnas.80.3.790 16578767 PMC393465

[B64] McNaughton S. J. Tarrants J. L. McNaughton M. M. Davis R. H. (1985). Silica as a defense against herbivory and a growth promotor in african grasses. *Ecology* 66 528–535. 10.2307/1940401

[B65] Melzer S. E. Chadwick O. A. Hartshorn A. S. Khomo L. M. Knapp A. K. Kelly E. F. (2011). Lithologic controls on biogenic silica cycling in South African savanna ecosystems. *Biogeochemistry* 108 317–334. 10.1007/s10533-011-9602-2

[B66] Melzer S. E. Knapp A. K. Kirkman K. P. Smith M. D. Blair J. M. Kelly E. F. (2009). Fire and grazing impacts on silica production and storage in grass dominated ecosystems. *Biogeochemistry* 97 263–278. 10.1007/s10533-009-9371-3

[B67] Mendoza M. Palmqvist P. (2008). Hypsodonty in ungulates: an adaptation for grass consumption or for foraging in open habitat? *J. Zool.* 274 134–142. 10.1111/j.1469-7998.2007.00365.x

[B68] Mitani N. Yamaji N. Feng Ma J. (2009). Identification of maize silicon influx ransporters. *Plant Cell Physiol.* 50 5–12. 10.1093/pcp/pcn110 18676379 PMC2638714

[B69] Morton B. B. Jutras M. W. (1974). Silica concentrations in grazed and ungrazed forage species. *Agron. J.* 66 10–12. 10.2134/agronj1974.00021962006600010003x

[B70] Oyarzabal M. Oesterheld M. (2009). Phosphorus reserves increase grass regrowth after defoliation. *Oecologia* 159 717–724. 10.1007/s00442-008-1263-z 19132398

[B71] Pahkala K. Pihala M. (2000). Different plant parts as raw material for fuel and pulp production. *Ind. Crops Prod.* 11 119–128. 10.1016/S0926-6690(99)00050-3

[B72] Piperno D. R. Pearsall D. M. (1998). The silica bodies of tropical american grasses: morphology, taxonomy, and implications for grass systematics and fossil phytolith identification. *Library* 85 1–40

[B73] Prasad V. Strömberg C. A. E. Alimohammadian H. Sahni A. (2005). Dinosaur coprolites and the early evolution of grasses and grazers. *Science* 310 1177–1180. 10.1126/science.1118806 16293759

[B74] Querné J. Ragueneau O. Poupart N. (2012). In situ biogenic silica variations in the invasive salt marsh plant, spartina alterniflora: a possible link with environmental stress. *Plant Soil* 352 157–171. 10.1007/s11104-011-0986-5

[B75] R Development Core Team. (2014). *R: A Language and Environment for Statistical Computing*, R Foundation for Statistical Computing, Vienna. Available at: http://www.R-project.org/

[B76] Raven J. A. (1983). The transport and function of silicon in plants. *Biol. Rev. Cambridge Philisophical Society* 58 179–207. 10.1111/j.1469-185X.1983.tb00385.x

[B77] Reynolds J. J. Lambin X. Massey F. P. Reidinger S. Sherratt J. A. Smith M. J. (2012). Delayed induced silica defences in grasses and their potential for destabilising herbivore population dynamics. *Oecologia* 170 445–456. 10.1007/s00442-012-2326-8 22526942

[B78] Rudall P. J. Prychid C. J. Gregory T. (2014). Epidermal patterning and silica phytoliths in grasses: an evolutionary history. *Bot. Rev.* 80 59–71. 10.1007/s12229-014-9133-3

[B79] Sangster A. G. Hodson M. J. Tubb H. J. (2001). “Silicon deposition in higher plants,” in *Silicon in Agriculture* eds Datnoff L. E. Snyder G. H. Korndorfer G. H. (New York: Elsevier), 85–113. 10.1016/S0928-3420(01)80009-4

[B80] Sangster A. G. Parry D. W. (1970). Silica deposition in the grass leaf in relation to transpiration and the effect of dinitrophenol. *Ann. Bot.* 35 667–677

[B81] Sanson G. D. Kerr S. A. Gross K. A. (2007). Do silica phytoliths eally wear mammalian teeth? *J. Archaeol. Sci.* 34 526–531. 10.1016/j.jas.2006.06.009

[B82] Schaller J. Brackhage C. Dudel E. G. (2012). Silicon availability changes structural carbon ratio and phenol content of grasses. *Environ. Exp.* *Bot.* 77 283–287. 10.1016/j.envexpbot.2011.12.009

[B83] Sinclair A. R. E. Norton-Griffiths M. (1979). *Serengeti: Dynamics of an Ecosystem*. Chicago, IL: University of Chicago Press

[B84] Soininen E. M. Bråthen K. A. Jusdado J. G. H. Reidinger S. Hartley S. E. (2013). More than herbivory: levels of silica-based defences in grasses vary with plant species, genotype and location. *Oikos* 122 30–41. 10.1111/j.1600-0706.2012.20689.x

[B85] Stebbins G. L. (1972). “The evolution of the grass family,” in *The Biology and Utilization of Grasses* eds Youngner V. B. McKell C. M. (New York: Academic Press).

[B86] Street J. R. (1974). *The Influence of Silica Concentration on the Chemical Composition and Decomposition Rates of Turfgrass Tissue and Water Absorption Rates among Three Turfgrass Species.* Ohio State University

[B87] Strömberg C. A. E. (2006). Evolution of hypsodonty in equids: testing a hypothesis of adaptation. *Paleobiology* 32 236–258. 10.1666/0094-8373(2006)32[236:EOHIET]2.0.CO;2

[B88] Strömberg C. A. E. (2011). Evolution of grasses and grassland ecosystems. *Annu. Rev. Earth Planet. Sci.* 39 517–544. 10.1146/annurev-earth-040809-152402

[B89] Strömberg C. A. E. Dunn R. E. Madden R. H. Kohn M. J. Carlini A. A. (2013). Decoupling the spread of grasslands from the evolution of grazer-type herbivores in South America. *Nat. Commun.* 4 1478. 10.1038/ncomms2508 23403579

[B90] Strömberg C. A. E. Werdelin L. Friis E. M. Saraç G. (2007). The spread of grass-dominated habitats in turkey and surrounding areas during the cenozoic: phytolith evidence. *Palaeogeogr. Palaeoclimatol. Palaeoecol.* 250 18–49. 10.1016/j.palaeo.2007.02.012

[B91] Struyf E. Van Damme S. Gribsholt B. Meire P. (2005). Freshwater marshes as dissolved silica recyclers in an estuarine environment (Schelde Estuary, Belgium). *Hydrobiologia* 540 69–77. 10.1007/s10750-004-7104-0

[B92] Van der Vorm P. D. J. (1980). Uptake of Si by five plant species, as influenced by variations in Si-supply. *Plant Soil* 56 153–156. 10.1007/BF02197962

[B93] Veldhuis M. P. Howison R. A. Fokkema R. W. Tielens E. Olff H. (2014). A novel mechanism for grazing lawn formation: large herbivore-induced modification of the plant–soil water balance. *J. Ecol.* 10.1111/1365-2745.12322

[B94] White F. (1983). *The Vegetation of Africa.* Paris: UNESCO

[B95] Wright I. J. Reich P. B. Westoby M. Ackerly D. D. Baruch Z. Bongers F. (2004). The worldwide leaf economics spectrum. *Nature* 428 821–827. 10.1038/nature02403 15103368

[B96] Zhu Z. Wei G. Li J. Qian Q. Yu J. (2004). Silicon alleviates salt stress and increases antioxidant enzymes activity in leaves of salt-stressed cucumber (*Cucumis Sativus* L.). *Plant Sci.* 167 527–533. 10.1016/j.plantsci.2004.04.020

